# Repurposing Drugs to Fight Hepatic Malaria Parasites

**DOI:** 10.3390/molecules25153409

**Published:** 2020-07-28

**Authors:** Diana Fontinha, Isabel Moules, Miguel Prudêncio

**Affiliations:** Instituto de Medicina Molecular João Lobo Antunes, Faculdade de Medicina, Universidade de Lisboa, Av. Prof. Egas Moniz, 1649-028 Lisboa, Portugal; dfontinha@medicina.ulisboa.pt (D.F.); isabel.moules@medicina.ulisboa.pt (I.M.)

**Keywords:** drug repurposing, malaria, *Plasmodium*, anti-plasmodial strategies, liver stage, pre-erythrocytic

## Abstract

Malaria remains one of the most prevalent infectious diseases worldwide, primarily affecting some of the most vulnerable populations around the globe. Despite achievements in the treatment of this devastating disease, there is still an urgent need for the discovery of new drugs that tackle infection by *Plasmodium* parasites. However, de novo drug development is a costly and time-consuming process. An alternative strategy is to evaluate the anti-plasmodial activity of compounds that are already approved for other purposes, an approach known as drug repurposing. Here, we will review efforts to assess the anti-plasmodial activity of existing drugs, with an emphasis on the obligatory and clinically silent liver stage of infection. We will also review the current knowledge on the classes of compounds that might be therapeutically relevant against *Plasmodium* in the context of other communicable diseases that are prevalent in regions where malaria is endemic. Repositioning existing compounds may constitute a faster solution to the current gap of prophylactic and therapeutic drugs that act on *Plasmodium* parasites, overall contributing to the global effort of malaria eradication.

## 1. Introduction

Malaria remains one of the most prevalent infectious diseases worldwide, constituting a particularly major health concern across Africa, Asia, and the Americas [1]. According to the World Health Organization (WHO), in 2018 alone there were an estimated 228 million cases of malaria worldwide, which resulted in 405,000 deaths [2]. Approximately 93% of the cases and deaths occurred in the WHO African Region, which carries the highest burden of malaria morbidity [2]. Children five years old and under are the most vulnerable group, representing 67% of all malaria deaths worldwide [2].

Malaria is caused by apicomplexan parasites of the *Plasmodium* genus, which are transmitted to the mammalian host through the bite of an infected female *Anopheles* mosquito. Five different *Plasmodium* species can cause malaria in humans: *P. falciparum*, *P. vivax*, *P. ovale*, *P. malariae*, and *P. knowlesi*. *P. falciparum*, which is responsible for most malaria cases and deaths, is prevalent in Africa, while *P. vivax* is more common in tropical areas outside of Africa, such as the Indo-Pacific and American regions. Although less deadly than *P. falciparum*, *P. vivax* and *P. ovale* have the ability to form hypnozoites, parasite forms that remain dormant in the liver and that can reactivate weeks, months, or even years after the initial mosquito bite, causing malaria relapses, which poses an additional challenge to disease eradication [3,4].

The life cycle of *Plasmodium* parasites is extremely complex, alternating between a mammalian and an invertebrate host, and comprising three developmental stages: the pre-erythrocytic or liver stage, the erythrocytic or blood stage, and the sporogonic or mosquito stage. When an infected female *Anopheles* mosquito takes a blood meal, *Plasmodium* sporozoites, the liver-infective form of the malaria parasite, are injected into the dermis of a mammalian host. Sporozoites use their gliding motility to reach the circulatory system and quickly access the liver [4,5]. After traversing the liver sinusoids through Kupffer and endothelial cells, the parasites traverse several hepatocytes before productively invading a final one [5,6,7]. During invasion, a parasitophorous vacuole (PV) is formed, inside which the parasite’s development into exoerythrocytic forms (EEFs) takes place. EEFs undergo a phase of asexual reproduction known as schizogony, thereby generating thousands of new parasites, termed merozoites. The pre-erythrocytic stage of infection culminates in the release and subsequent bursting of merosomes, merozoite-filled vesicles containing around 40,000 merozoites per infected hepatocyte, into the bloodstream [4,5,8]. Free merozoites then infect erythrocytes, and undergo the successive cycles of invasion, intracellular growth, proliferation, and re-invasion that constitute the erythrocytic stage of infection. Within the intra-erythrocytic PV, merozoites go through different stages of development with distinct morphologies, termed the ring, trophozoite, and schizont stages [9]. A proportion of blood stage parasites undergoes a developmental switch, initiating their commitment to sexual development, a process known as gametocytogenesis, which is crucial for malaria transmission [5]. When a female *Anopheles* mosquito feeds from an infected mammalian host, she ingests male and female gametocytes, initiating the sporogonic stage of the parasite’s life cycle. Gametocytes develop into gametes that fuse in the mosquito midgut, forming a zygote, which then transforms into an ookinete that develops into an oocyst [5]. Asexual sporogonic replication occurs and thousands of motile sporozoites invade the salivary glands of the mosquito, where they remain ready to be injected into a new mammalian host [5].

**Figure 1:**
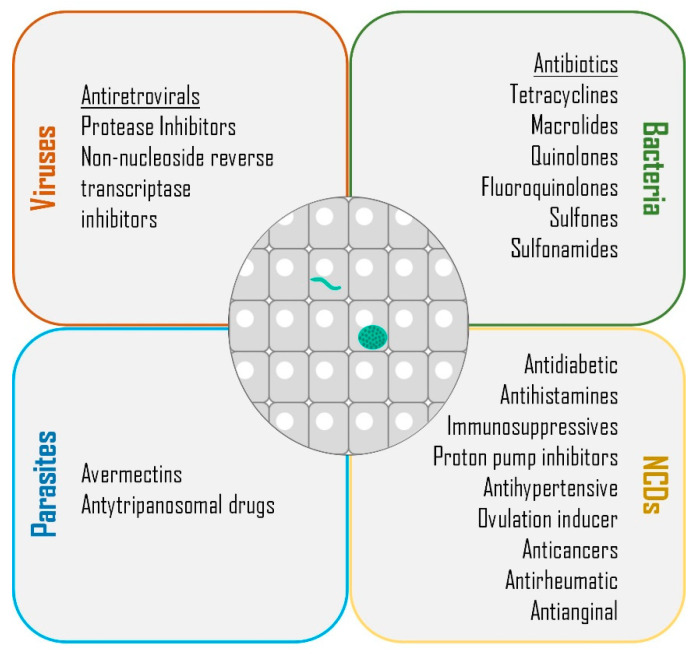
Classes of drugs with demonstrated liver stage activity. Summary of the classes of compounds employed in the management of viral, bacterial, parasitic, and non-communicable or chronic diseases (NCDs) that have been demonstrated to impact the liver stage of *Plasmodium* parasites.

Despite extensive efforts to combat malaria, the reduction of this disease’s global incidence rate has halted since 2015, and the decrease of the mortality rate has slowed since 2016 [2]. Throughout the years, multiple efforts have been undertaken towards the development of an effective vaccine, an ambitious goal that remains to be fulfilled. In the absence of a vaccine, the WHO recommends vector control to reduce the chances of mosquito bites, and chemoprevention to suppress infection [2]. As the current pipeline of anti-plasmodial drugs is unlikely to be sufficient to progress the malaria elimination agenda, it has become important to discover new therapeutics with broad activity to eliminate asexual pre-erythrocytic and erythrocytic parasites, clear pre-erythrocytic hypnozoites, and block parasite transmission [5]. Besides providing an opportunity for the radical cure of hypnozoite-forming species, the obligatory and clinically silent nature of the liver stage of *Plasmodium* infection make it an attractive target for pharmacologic prophylaxis. However, the specificities of sporozoite production, which requires access to an insectarium, limit the drug screening efforts against this step of infection, making them less frequent than those aimed at assessing blood stage activity. Furthermore, since drug development is costly and time-consuming, alternative approaches are necessary to streamline the process of discovery of novel malaria therapeutics. Drug repurposing, also known as drug repositioning, reprofiling, or re-tasking, is a strategy aimed at the identification of new uses for approved or investigational drugs that are outside the field of reference of the original medical indication [10,11]. Here, we will review the possibility of repurposing drugs employed in the management of various viral, bacterial, parasitic, and non-communicable diseases (NCDs) against malaria parasites, with an emphasis on the pre-erythrocytic bottleneck of *Plasmodium* infection (Figure 1).

## 2. Drug Repurposing

Drug repurposing offers many advantages over developing an entirely new drug for a given indication. First, and perhaps most importantly, the risk of failure of a repositioned drug is lower than that of one developed de novo, because the safety assessment of the former has already been completed in preclinical models and in humans. Second, the time frame for drug development is reduced because most of the preclinical testing and safety assessment have already been performed. Moreover, in some cases, formulation development will have been completed. This results in an estimated 3–12-years process for repurposing existing drugs versus a 10–17-years process for de novo drug development [10,12]. Third, from an economical perspective, bypassing the development stages is estimated to reduce around 15% of the overall costs [12,13,14]. Drug repurposing has, therefore, become a popular and advantageous approach in recent years, circumventing much of the discovery and preclinical stages, since pharmacokinetic, pharmacodynamic, and toxicity profiles of the drugs are often already known, reducing time, cost, and investment, and decreasing the risks of failure [12,15,16].

Various drugs have been successfully repurposed throughout the years, as is the case of auranofin, thalidomide, and zidovudine. Auranofin, a Food and Drug Administration (FDA)-approved drug, employed in the treatment of rheumatoid arthritis for 25 years, was identified in a High Throughput Screening (HTS) and repurposed for the treatment of human amoebiasis, a protozoan intestinal parasitic disease caused by *Entamoeba histolytica*, which is responsible for 100,000 deaths globally per year [12,17,18]. Auranofin’s potency against this pathogen was ten-fold higher than that of the control drug, metronidazole [17]. In 2012, FDA granted auranofin the orphan drug status for the treatment of human amoebiasis [12].

Thalidomide was originally marketed in Germany and England as a sedative, and was specifically prescribed to treat morning sickness in pregnant women [10]. Taking the drug as indicated in the first trimester of pregnancy resulted in severe skeletal birth defects in at least 15,000 children, causing the drug to be banned from the market [10]. However, thalidomide was later repurposed for the treatment of erythema nodosum granulosis (ENL), a severe inflammatory condition of leprosy [10,19]. Thalidomide was first approved by the FDA in 1998 for cutaneous manifestations of ENL in leprosy [10], and remains the only drug used to treat this condition [10].

Originally developed for cancer treatment in 1964, zidovudine (AZT), was approved for the treatment of Human Immunodeficiency Virus (HIV)/Acquired ImmunoDeficiency Syndrome (AIDS) in March 1987, few years after the first report of AIDS and its etiological infectious agent, HIV [20,21]. The combination of fundamental research, regulatory advantages, drug repurposing and, collaboration with HIV/AIDS advocate groups was essential to move this drug forward as an antiretroviral suitable for clinical use [20].

Besides the drugs that have already been successfully repurposed, several other candidates are presently under consideration. Such is the case of chlorcyclizine, an over-the-counter drug for allergy symptoms first approved in 1940, which may be repurposed for the treatment of Hepatitis C Virus (HCV) infection [12,22]. The number of people affected worldwide by this disease, estimated to be 185 million, creates an unmet need for the development of new effective and affordable therapeutic options [12]. Chlorcyclizine exerts its antiviral effect at an early stage of HCV infection, possibly by targeting viral entry into the host cells [12], without evidence of emerging drug resistance. Collectively, these observations put forth chlorcyclizine as a promising candidate for drug repurposing for the treatment of HCV infection [12,22].

The anti-plasmodial strategies toolbox has also benefitted from a successful case of drug repurposing, the tetracycline antibiotic doxycycline, which impacts both pre-erythrocytic and erythrocytic *Plasmodium* parasites, as will be described in detail below. In fact, doxycycline is currently recommended by the Centers for Disease Control and Prevention (CDC) as a chemoprophylaxis option for travelers, as well as for the treatment of uncomplicated malaria in combination with other anti-plasmodial drugs, but it is contraindicated in pregnant women and in children under the age of 8 [23]. Besides doxycycline, many other drugs not primarily aimed for the management of *Plasmodium* infection have been screened for their anti-plasmodial activity. Their impact on the liver stage of infection and/or on other parasite stages and their consequent potential for repurposing towards malaria will be the focus of this review.

## 3. Targeting the Liver Stage of *Plasmodium* Infection

The clinically silent liver stage of *Plasmodium* infection that takes place in the mammalian host is a major bottleneck of the malaria parasite’s life cycle. Hepatic infection obligatorily precedes the blood stage of infection, where disease takes place, providing an opportunity for targeted prophylaxis [4]. Furthermore, in the case of relapsing *Plasmodium* species, the liver also constitutes a reservoir of dormant hypnozoites that can reactivate weeks to years after the primary infection, posing an additional burden to the global effort of malaria eradication [24]. Therefore, targeting this stage of the parasite’s life cycle is an absolute requirement to achieve the radical cure of infection by relapsing *Plasmodium* parasite species. Currently, only two drugs, primaquine and tafenoquine, have been approved for the elimination of hypnozoites in the treatment of malaria relapses [25]. However, the use of these 8-aminoquinolines is hampered by their toxicity. In fact, these drugs are known to cause hemolysis in patients with glucose-6-phosphate dehydrogenase (G6PD) deficiency, a genetic disorder that is fairly common in malaria endemic areas [26]. Hence, the existing gap in the prophylactic and therapeutic needs related to hepatic infection by *Plasmodium* must be urgently fulfilled. One possible approach to this challenge lies in exploiting the possibility of repurposing approved drugs from various therapeutic categories to bridge this gap (Table 1).

### 3.1. From HIV to Parasites

*Plasmodium* parasites and HIV share a geographical presence in several regions worldwide. In this context, antiretroviral (ARV) compounds, employed in the treatment of HIV infection, have been assessed for their activity against *Plasmodium* liver stage in vitro and in vivo, in both rodent and human models of malaria. Among the classes of compounds employed in ARV therapy, protease inhibitors (PIs) have been the most extensively evaluated against *Plasmodium* parasites. In particular, the PIs saquinavir, lopinavir, ritonavir, and indinavir were shown to act upon the hepatic stages of the rodent parasites *P. yoelii* [27] and/or *P. berghei* [37]. Lopinavir was further shown to impact the liver stage of the human-infective *P. falciparum* parasite at clinically-relevant concentrations [36]. Lopinavir/ritonavir displayed dose-dependent in vivo activity against developing hepatic *P. yoelii* parasites [37], whereas the non-nucleoside reverse transcriptase inhibitors (NNRTIs) efavirenz, etravirine, and nevirapine only modestly impacted this parasite’s liver load [42]. In another study, efavirenz, etravirine, and the PI nelfinavir were shown to impair *P. berghei*’s intra-hepatic development in a human hepatoma cell line. The field combinations recommended by the WHO, specifically efavirenz + AZT + lamivudine, efavirenz + tenofovir + emtricitabine, and nevirapine + tenofovir + emtricitabine [131], were additionally evaluated in an in vivo setting, resulting in a reduction of parasite liver load, which could be further enhanced by the replacement of efavirenz by etravirine [38]. All PIs that display activity against hepatic infection by *Plasmodium* parasites have also been tested in vitro and/or in vivo against the ensuing blood stage of infection of *P. berghei*, *P. cynomolgi*, *P. knowlesi*, *P. vivax*, and *P. falciparum* [28,29,30,31,32,33,34,39,40,43], with lopinavir appearing to display the strongest activity among this class of compounds. Besides impacting asexual blood stages, the PIs lopinavir, saquinavir, and ritonavir also inhibited gametocytogenesis of *P. falciparum* parasites [33]. This was further confirmed by a second study that demonstrated that lopinavir and saquinavir reduced gametocyte viability and displayed *P. falciparum* transmission blocking activity [35]. Lopinavir and ritonavir were further reported to reduce ookinete and oocyst formation by *P. berghei*, while ritonavir, saquinavir, and indinavir were shown to inhibit this parasite’s oocyst development [132]. The activity of PIs against *Plasmodium* parasites has been suggested to be mediated by the inhibition of their aspartyl proteases, some of which localize to the food vacuole and are involved in the degradation of haemoglobin [28,133,134,135,136]. The assessment of the activity of the NNRTIs efavirenz, etravirine, and nevirapine against the blood stage of *P. falciparum* revealed that the latter is not effective [31,34]. Further analysis of nevirapine in the context of an in vivo *P. berghei* infection showed only a modest impact in the control of parasitaemia [43]. Once in the insect vector step of the parasite’s life cycle, the NNRTI etravirine displayed activity against all sporogonic stages, whereas efavirenz only impacted oocyst formation and nevirapine was inactive [132]. Besides in vitro and in vivo testing against the different stages that compose the *Plasmodium* parasite’s life cycle, ARV compounds have also been assessed in the field, in both children and adult populations, as briefly reviewed by Hobbs et al. [137]. The broad activity demonstrated by some ARV compounds against the liver, blood, and sporogonic stages of *Plasmodium* infection sets the stage for their repositioning as possible multistage anti-plasmodial strategies. The co-endemicity of HIV and malaria further supports the need to deeply understand the effect of ARV compounds on *Plasmodium* infection. Such knowledge may be employed to adjust the current ARV treatment recommendations to gain a bonus anti-plasmodial effect without compromising the control of HIV infection.

### 3.2. From Bacteria to Parasites

The repurposing of various classes of antibiotics employed in the management of bacterial infections to be used against the liver stage of *Plasmodium* infection has been extensively investigated. Among the antibiotics most widely studied in this regard are tetracyclines, macrolides, quinolones, fluoroquinolones, sulfones, and sulfonamides.

The broad-spectrum antibiotic tetracyclines have been shown to impact the liver stage of *Plasmodium* infection. Early in vivo studies demonstrated that demeclocycline extended the prepatent period of *P. cynomolgi* infection in rhesus monkeys, while further displaying blood schizonticidal activity [44]. Terramycin and minocycline were also shown to impact the liver stage of rhesus infection by *P. cynomolgi* in vivo and/or the liver stage of *P. berghei* in vitro [45,46,47]. The latter compound further displayed activity against the blood stages of this parasite [48,49,50,51], although another study suggested that parasite resistance to this compound could emerge [52]. Nevertheless, within the tetracycline antibiotics class, the synthetically derived doxycycline is the one that has been most widely investigated for anti-plasmodial intervention, and was recommended for chemoprophylaxis of malaria as early as 1985 [138,139]. Since then, doxycycline has been evaluated against the liver stage of *P. berghei* and *P. yoelii* parasites, and was shown to reduce hepatic parasite numbers and development in vitro, and completely prevent the ensuing parasitemia in vivo [54]. Furthermore, its efficacy as a prophylactic agent has been validated in several clinical trials, the failures reported in these studies having mostly been associated with inadequate dosage or poor patient compliance, as extensively reviewed by Gaillard et al. [140]. Doxycycline was also shown to target the *P. falciparum* apicoplast [55,56], impacting the parasite’s erythrocytic forms [48,55,57,58]. The compound’s slow-acting activity justifies the need to combine doxycycline with a faster-acting compound to target this stage of infection.

The potential application of the macrolide antibiotic azithromycin as a prophylactic agent was initially proposed based on the protection observed in a mouse model of *P. yoelii* infection [59]. This activity was later ascertained in clinical trials employing different drug administration regimens and against different parasite species, specifically *P. falciparum* [141,142,143,144] and *P. vivax* [145]. Azithromycin was found to be less effective than doxycycline against *P. falciparum* parasites, but both compounds were equally active in a *P. vivax*-endemic region [143,145]. Despite its lower activity against *P. falciparum*, azithromycin may be useful to treat individuals with contraindications to the administration of doxycycline, such as pregnant women and children under the age of 8. In fact, the combination of azithromycin with chloroquine has been proposed as an alternative for intermittent preventive treatment of malaria in pregnancy, as reviewed by Chico et al. [146]. Further detailed studies in the mouse model of *P. berghei* infection revealed that azithromycin did not impact the number nor the development of intrahepatic parasites, but instead rendered the liver-derived merozoites non-infectious [60]. Azythromycin was also shown to impact the blood stage of infection of *P. berghei* or *P. falciparum* [48,61,62,63]. Similar to doxycycline, it behaves as a slow-acting drug that targets the parasite’s apicoplast [56,61,64,147]. Consequently, its activity against the blood stage of *Plasmodium* infection may benefit from the combination with faster acting compounds, an option that has been exploited in clinical trials, as reviewed by Rosenthal et al. [148]. Besides the impact that it exerts on the life cycle of the parasite in the mammalian host, azithromycin was further shown to inhibit *P. berghei* development in the insect vector [147].

A panel of 25 quinolones and fluoroquinolones was screened in vitro for activity against *P. yoelii* hepatic infection, revealing that some members of this class of broad-spectrum antibiotics have potential use as prophylactic agents for malaria. Among the 25 compounds tested, grepafloxacin, norfloxacin, piromidic acid, trovafloxacin, cinoxacin, ciprofloxacin, rufloxacin, sparfloxacin, ofloxacin, temafloxacin, pefloxacin, and clinafloxacin displayed variable anti-plasmodial activity, with IC_50_ values ranging between 4.4 and 90.2 µg/mL. Grepafloxacin, piromidic acid, and trovafloxacin were among the most active compounds, impacting not only parasite numbers but also its development and morphology. These compounds were additionally shown to display a similar activity profile against the in vitro infection of primary human hepatocytes by *P. falciparum*. Furthermore, all 25 compounds, most notably grepafloxacin, trovafloxacin, and ciprofloxacin, displayed in vitro activity against the blood stage of chloroquine-sensitive and chloroquine-resistant *P. falciparum* strains [65]. These observations were in agreement with other reports of blood stage anti-plasmodial activity of quinolones and fluoroquinolones [48,66,67,68,69]. In a mouse model of *P. yoelii* infection, ciprofloxacin only modestly decreased parasitaemia, but prevented mortality [70]. Ciprofloxacin’s blood stage activity increased with prolonged exposure [66,69,71] and has been linked to the formation of an abnormal apicoplast [56].

The sulfone antibiotic dapsone, also known as diaminodiphenyl sulfone, which is used in combination in the treatment of leprosy, was shown to exhibit mild activity against the liver stage of *P. yoelii* in a mouse model of infection, as estimated from the appearance of ensuing blood parasitemia [72]. Dapsone was combined with pyrimethamine (Maloprim, GlaxoSmithKline) or with proguanil for malaria prevention, but these combinations were eventually withdrawn due to hematological side effects [149,150]. Besides its use in prophylaxis, dapsone was further combined with chlorproguanil (LapDap, GlaxoSmithKline) for the treatment of malaria, which was also withdrawn in 2008 due to safety issues in G6PD-deficient patients [150,151].

Co-trimoxazole, a combination of the dihydrofolate reductase inhibitor trimethoprim and the sulfonamide antibiotic sulfamethoxazole (TMP-SMX), is employed in the prevention of opportunistic infections, including clinical episodes of *P. falciparum* malaria, in HIV-infected patients. In vitro, TMP-SMX inhibited the intra-hepatic development of rodent *P. berghei* and *P. yoelii* parasites and reduced the numbers of hepatic *P. falciparum* parasites. Accordingly, this drug impacted the *P. yoelii* liver load in a mouse model of infection, preventing the subsequent appearance of parasitemia [42]. TMP-SMX was later tested in a non-human primate model of infection, where it inhibited the intra-hepatic development of *P. knowlesi* parasites in rhesus macaque livers [73]. TMP-SMX was also active against the blood stage of *P. falciparum* infection in vitro [74,75]. In humans, TMP-SMX has been assessed and proven effective in many studies for the treatment and prophylaxis of malaria, as extensively reviewed by Manyando et al. [152] and by Gaillard et al. [153]. Another sulfonamide antibiotic, sulfadiazine, displayed mild activity against hepatic infection by *P. yoelii* [72] and *P. berghei* [76] in vivo. Sulfadiazine was also shown to impact early erythrocytic schizonts of *P. berghei* and *P. cynomolgi* in vivo as early as 1951 [77,78]. When tested against *P. falciparum* blood stages in combination with pyrimethamine, sulfadiazine was inferior to sulfadoxine [79], which is currently part of the WHO recommendations for intermittent preventive treatment for pregnant women and infants [154].

As illustrated by the successful case of doxycycline, antibiotics have great potential for repurposing against *Plasmodium* parasites. Their action may either be stage-specific or may impact the parasite throughout infection of the mammalian host. Given the limited availability of *Plasmodium* sporozoites, many studies have solely focused on the identification of antibiotics with blood stage activity. In the future, the screening of those drugs against *Plasmodium* hepatic infection will likely identify additional antibiotics with multistage anti-plasmodial activity.

### 3.3. Repurposing within Parasitism

Avermectins are a family of macrocyclic lactones with antiparasitic and insecticidal properties that have already been proposed for the reduction of the incidence of vector-borne diseases [155]. Within this family, ivermectin has been employed in mass drug administrations to treat neglected tropical diseases, namely onchocerciasis and lymphatic filariasis (LF) [156,157]. Four compounds of this class, ivermectin, abamectin, emamectin, and eprinomectin, were shown to impair the intra-hepatic development of *P. berghei* in vitro [47,80]. Ivermectin further reduced the number of parasites present in the in vivo mouse liver, impacting both parasite development and host survival [80]. A cluster-randomized clinical trial aimed at assessing the impact of repeated ivermectin mass drug administrations on malaria control revealed that this compound could reduce the incidence of malaria episodes in children by 20% [158]. The applicability of ivermectin for causal malaria prophylaxis was further evaluated in a controlled human malaria infection trial, in which 8 volunteers received a single oral dose of 0.4 mg/kg of ivermectin, two hours prior to *P. falciparum* sporozoite injection. At the dose and regimen employed, ivermectin did not impact pre-patency [159]. The activity of ivermectin against hepatic *Plasmodium* infection added up to its previously known impact against this parasite’s life cycle from the mammalian host through to the insect vector. In the former, ivermectin impacted the asexual blood stages of *P. falciparum* [81,82] and, to some extent, mature gametocytes [81]. In the mosquito stage of the parasite’s life cycle, ivermectin not only impacted the sporogony of the parasite [160,161,162,163], but also controlled the mosquito population [164,165,166,167,168,169,170]. Ivermectin can impact transmission [171,172,173] and has been suggested as a tool for malaria control [158,174,175,176,177]. Overall, the generalized activity of ivermectin upon the various aspects of *Plasmodium* infection make it a very promising tool in the prevention, treatment, and blocking of parasite transmission.

Human African trypanosomiasis (HAT), also known as sleeping sickness, is caused by protozoan *Trypanosoma brucei gambiense* and *T. b. rhodesiense* parasites, which are transmitted by tsetse flies. This disease poses a threat to 36 countries in sub-Saharan Africa and is, therefore, co-endemic with malaria. HAT is characterized by two disease stages, for which there are alternative treatments. For the first stage, when parasites replicate in subcutaneous tissues, blood, and lymph, treatment recommendations include pentamidine and suramin. In the second stage, when parasites have crossed the blood–brain barrier to infect the central nervous system, treatment recurs to melarsoprol, eflornithine, and nifurtimox. In 2019, fexinidazole was included in the WHO Essential Medicines List (EML) and WHO’s treatment guidelines for both first stage and non-severe second stage HAT cases [178,179]. Suramin inhibits the binding of thrombospondin related anonymous protein (TRAP) to the surface of HepG2 cells, consequently inhibiting *P. falciparum* invasion of these hepatocytes with an IC_50_ of 50 µM [83]. Besides its liver stage activity, suramin was also shown to inhibit *P. falciparum* invasion of erythrocytes, possibly by binding to and preventing the proteolytic cleavage of merozoite surface protein-1 (MSP1) [84]. Suramin was further shown to impact *P. berghei* parasitaemia in vivo, as well as *P. falciparum* intraerythrocytic development in vitro [85]. Besides TRAP and MSP1, suramin has been further associated with other *P. falciparum* targets. Specifically, it was shown to inhibit the catalytic activity of a parasite aldolase [86], as well as of a recombinant 3-phosphoglycerate kinase [87], both of which are part of the glycolytic pathway. Suramin was also proven to bind to recombinant falcipain-2 and to inhibit its cysteine protease activity, a key step in the hydrolysis of hemoglobin by this parasite during the erythrocytic stage of its life cycle [88]. Finally, suramin was shown to weakly inhibit *P. falciparum* diadenosine tetraphosphate hydrolase [89]. However, its nonspecific interactions and consequent toxicity hinder its use as an anti-plasmodial compound. Eflornithine, also known as α-difluoromethylornithine (DFMO), is an irreversible inhibitor of ornithine decarboxylase, the rate-limiting enzyme of polyamine synthesis [180]. This compound was shown to impact the exoerythrocytic stage of *P. berghei* in vivo in a dose-dependent manner, as assessed by the presence or absence of an ensuing parasitaemia. In this model, DFMO had no impact on the parasite’s erythrocytic stages [90]. DFMO’s activity against hepatic stage rodent malaria parasites was further demonstrated both in vivo [91,92,93,94] and in vitro in *P. berghei*-infected human hepatoma cells [92]. Contrary to previous observations, DFMO was later reported to limit the erythrocytic schizogony of *P. berghei* in vivo [91,95,96], although it did not improve the survival of the treated mice [95]. When incubated in vitro with blood stage cultures of *P. falciparum*, DFMO impacted parasitaemia in the millimolar range [95,97,98,99,100,101,102], preventing the transformation of trophozoites into schizonts [98,101]. Moreover, this drug was shown to inhibit the sporogonic cycle of *P. berghei* in the *Anopheles stephensi* vector [181]. Pentamidine, another drug employed in the management of HAT, has never been tested against hepatic *Plasmodium* infection. Nonetheless, it exhibited nanomolar-ranged activity against in vitro *P. falciparum* blood stage [103,104,105,106,107,108,109,110,111]. This drug was shown to be selectively taken up by *P. falciparum*-infected erythrocytes, where it bound to the toxic ferriprotoporphyrin IX generated during hemoglobin digestion, inhibiting its crystallization into the non-toxic hemozoin [112]. Pentamidine was also active against *P. berghei* blood stages in vitro, although at higher concentrations than against *P. falciparum* [113]. Melarsoprol, nifurtimox, and fexinidazole have not yet been assessed for anti-plasmodial activity. Further screening of these compounds may inform their repositioning against *Plasmodium* hepatic and/or blood stage infection.

### 3.4. From Chronicity to Infection

Although anti-infective medicines have been extensively tested for repurposing against *Plasmodium* parasites, other, perhaps less obvious, candidates include medicines that are employed in the treatment of non-communicable or chronic diseases. These comprise antidiabetic drugs, antihistamine agents, immunosuppressive compounds, proton pump inhibitors, antihypertensive agents, an ovulation inducer, anticancer drugs, an antirheumatic agent, and an antianginal agent.

Metformin, a biguanide antidiabetic drug that primarily targets the liver, was shown to inhibit hepatic infection in vivo in the *P. berghei* mouse model, where it decreased both parasite numbers and intra-hepatic parasite development. The latter effect was also observed in vitro employing a model of infection of primary human hepatocytes by the human malaria parasite, *P. falciparum* [114]. Despite its demonstrated activity against hepatic infection, metformin only modestly impacted asexual erythrocytic stages, as shown in vivo in two rodent models of infection, *P. berghei* and *P.yoelii*, and in vitro, using *P. falciparum* [114,115].

The potential prophylactic activity of the antihistamine agents cyproheptadine, ketotifen, terfenadine, azatadine, and loratadine, employed in the management of allergies, against a chloroquine-resistant strain of *P. yoelii* has been evaluated. Complete prevention of hepatic infection was achieved with cyproheptadine and ketotifen at 5 mg/kg, as well as for terfenadine at a higher dose of 50 mg/kg of mouse body weight. Azatadine and loratadine also demonstrated partial activity against the liver stage of infection. It should be noted that the impact of these drugs on hepatic infection was not assessed directly on liver samples but was rather estimated from the ensuing appearance of blood stage parasitemia. Nonetheless, all compounds were shown to be inactive against erythrocytic parasite forms at the concentrations that were effective for prophylaxis, confirming that the impact observed in pre-patency results solely from their activity against hepatic parasites [116]. In another study, cyproheptadine and ketotifen were shown to display low blood stage activity against *P. falciparum* in vitro [117], *P. berghei* in vivo [118], and *P. yoelii* in vivo [119]. Cyproheptadine, ketotifen, azatadine and loratadine were shown to increase mouse survival when administered as a treatment of *P. yoelii* infection [119]. Cyproheptadine was also effective against the blood stage of a multidrug-resistant strain of *P. yoelii* [120]. In humans, ketotifen was employed in combination with chloroquine and/or Fansidar (sulfadoxine-pyrimethamine; Roche), demonstrating its potential for the treatment of uncomplicated falciparum malaria [182], whereas it did not improve chloroquine’s anti-plasmodial activity in children [183]. Two additional antihistaminics, clemastine and astemizole, were later identified in a high-throughput phenotypic liver stage *P. berghei* parasite screen, and were further shown to be active against the *P. falciparum* blood stage [121]. Astemizole’s activity against the blood stage of *Plasmodium* infection is in agreement with a previous study where this compound impacted the erythrocytic infection of *P. falciparum* in vitro, as well as of chloroquine-sensitive *P. vinckei* and chloroquine-resistant *P. yoelii* in vivo [123]. Recently, a *Plasmodium* chaperonin TRiC/CCT was identified as the target of clemastine [122].

The immunosuppressive agent cyclosporin A, which is commonly used to inhibit the rejection of transplanted organs, displayed activity in the nanomolar range against *P. berghei* infection of a human hepatic cell line [121]. When administered in vivo, cyclosporin A increased the pre-patency time of *P. berghei* infection by 1.7 days [124]. Besides its prophylactic potential, cyclosporin A was also demonstrated to be useful as a therapeutic agent, as observed by its blood stage activity against *P. berghei*, *P. yoelii* and/or *P. chabaudi* in vivo [124,125,126,127], and against both *P. falciparum* [121,125] and, to a lesser extent, *P. vivax* [128] in vitro. This blood stage activity has been suggested to result from the permeabilization and aggregation of sphingomyelin-rich membranes that are formed by the parasite throughout this step of its life cycle [129,130].

Two HTS studies identified several medicines employed in the treatment of NCDs as having activity against the in vitro liver stage of *P. berghei*, namely the proton pump inhibitors omeprazole and esomeprazole; the angiotensin receptor antagonist telmisartan; the ovulation inducer clomiphene; the anticancer drugs daunorubicin, doxorubicin, and idarubicin; the antirheumatic agent auranofin; the antihistamine agent clemastine fumarate; the immunosuppressive agent mycophenolic acid; the structurally-related estrogen receptor modulators tamoxifen citrate and toremiphene citrate employed in the treatment of breast cancer; and the prophylactic antianginal agent perhexiline maleate (see [47,121] and references therein).

Perhaps surprisingly, several medicines for NCDs have been found to impact *Plasmodium* infection, an activity that spans throughout the parasite’s life cycle in the mammalian host. These observations support the notion that drug repurposing for an infectious disease should not be limited to in-kind compounds and that, instead, the options available for the development of repurposing strategies should be expanded to include other, arguably less obvious, compounds.

### 3.5. Targeting Parasite Dormancy

Besides prophylaxis, the liver stage of *Plasmodium* infection can also constitute a target for therapeutic interventions, in the case of relapsing parasite species that form dormant hypnozoites in the liver of the mammalian host. The laboratory models to directly address this issue are somewhat limited, which may help explain the scarcity of drug repurposing studies against this step of the parasite’s life cycle.

Tinidazole, an anti-infective drug that is used against protozoan infections, and triamterene, a diuretic drug that is employed in the management of hypertension, have been shown to moderately protect from relapse, in a model of rhesus macaque infection by *P. cynomolgi*. Tinidazole’s activity was improved when it was combined with chloroquine and low dose primaquine. The same was observed for pyrazinamide, a drug employed in the management of tuberculosis (TB), but not for the antihistamine drug promethazine, nor for the antibiotics clindamycin, minocycline, doxycycline, azithromycin, and ciprofloxacin [53]. Despite the promising observations in the *P. cynomolgi* model of infection, tinidazole was later proved to be ineffective in preventing relapse of *P. vivax*, in a pilot phase II clinical trial [184]. The protease inhibitor lopinavir-ritonavir and the antimicrobial TMP-SMX, which, as described above, have been shown to impact *Plasmodium* liver infection, were also assessed for their activity against *P. cynomolgi* hypnozoites, and shown to be ineffective in preventing malaria relapse in rhesus monkeys [41].

The development of new tools for straightforward hypnozoite identification, such as the recently developed dual-fluorescent [185] and dual-luminescent [186] *P. cynomolgi* reporter parasite lines, and the establishment and optimization of drug screening procedures for this step of infection, will streamline drug repurposing towards the elimination of dormant parasite forms, an obstacle to the global effort of malaria eradication.

## 4. Co-Endemic Infectious Diseases: An Opportunity for Repurposing

Malaria affects the poorest regions of the globe, where it poses huge health and economic challenges. These regions are also affected by many other infectious diseases, for which vaccination is not always available, and which may require recurrent treatment. Therefore, assessing the potential activity of the drugs employed in the management of such infectious diseases against *Plasmodium* infection may identify bonus effects or suggest alternative treatments that can tackle both conditions simultaneously. Furthermore, these drugs may be readily available in malaria-endemic regions, facilitating their employment against this disease. In many cases, such drugs already have reported blood stage activity, but their impact on *Plasmodium* hepatic infection remains elusive. Further characterization of these medicines may create the possibility for their employment as malaria prophylaxis and/or multistage anti-plasmodial strategies.

TB, a disease caused by infection with *Mycobacterium tuberculosis* bacteria, is the leading cause of death from a single infectious agent [187]. Although TB is spread worldwide, it mostly affects developing countries [187], where it overlaps with malaria. Standard treatment against TB requires a 6-month course of antimicrobial combinations. Given the long-term nature of the treatment, a bonus effect of such antituberculosis drugs against *Plasmodium* parasites could help control malaria in regions where both infections are co-endemic. In fact, most tuberculostatics have already been tested against the blood stage of *Plasmodium* infection. However, their effectiveness against the parasite’s liver stage bottleneck remains largely unknown. An exception to this rule is the panel of fluoroquinolones that belong to the second line of TB treatment, which have been tested in terms of their liver stage anti-plasmodial activity, as described above.

The first line treatment against TB relies on the combination of ethambutol, isoniazid, rifampicin, and pyrazinamide [188]. Except for pyrazinamide, the activity of these compounds against the blood stage of *P. berghei* infection has been assessed in vivo, revealing that only the combination of all three compounds, or of rifampicin and isoniazid, reduces blood parasitaemia and increases mouse survival [189]. The combination of rifampicin with isoniazid with the addition of TMP-SMX (Cotrifazid) was further evaluated in clinical trials, a combination that was initially shown to be suitable for the treatment of malaria [190,191,192], but was later proven to be inferior to mefloquine and to the combination of quinine with sulfadoxine-pyrimethamine against chloroquine- or amodiaquine-resistant malaria [193]. Isoniazid was also suggested to impact the sporogonic stage of *P. berghei* parasites, an effect that was observed in vivo, but not in vitro, suggesting an indirect mechanism of action [194]. Rifampicin in monotherapy also displayed activity against the blood stages of *P. berghei* in vivo, increasing mouse survival [195], against *P. chabaudi* in vivo, reducing parasitaemia, and against *P. falciparum* in vitro, reducing the viability of ring forms [196]. However, rifampicin was not effective when employed as a monotherapy against *P. vivax*-infected patients [197]. Additional in vitro assays confirmed rifampicin’s activity against the blood stage of *P. falciparum*’s life cycle [48,56,69,198,199].

Besides fluoroquinolones, other drugs employed as second-line TB treatment have also been screened for their blood stage anti-plasmodial activity, but their efficacy against the parasite’s liver stages remains unaddressed. Such is the case of the injectable agents amikacin, kanamycin, and streptomycin. Amikacin was shown to delay *P. falciparum*’s erythrocytic development, an activity that was probably linked to the inhibition of a parasite’s acid phospholipase [200]. Conversely, kanamycin and streptomycin were well tolerated in *P. falciparum* blood stage cultures and did not impact this phase of the parasite’s life cycle [199,201,202]. The oral bacteriostatic agent cycloserine was shown to impact *P. falciparum* erythrocytic growth at an IC_50_ that is close to that of *M. tuberculosis*, by acting upon the essential sulfur mobilization pathway required for apicoplast maintenance, which is of bacterial origin [203]. In silico analysis suggested that cycloserine may equally impact *P. vivax* blood stages by acting on the pathway that was demonstrated for *P. falciparum.* However, these observations still require experimental validation [204]. The macrolide clarithromycin was shown to reduce blood stage parasitaemia of multidrug-resistant *P. yoelii* [205], to disrupt the apicoplast of *P. falciparum* [206,207], and to have a synergistic activity in combination with mefloquine [205,206]. Another second-line TB treatment agent, clofazimine, displayed mild to no anti-plasmodial activity against the *P. falciparum* blood stage [208,209]. Linezolid had no effect on the growth of *P. falciparum* blood stage parasites [210], whereas thioridazine displayed only mild anti-plasmodial activity against the *P. falciparum* blood stages [211]. The anti-plasmodial activity of the tuberculostatic agents capreomycin, ethionamide, para-aminosalicylic acid, prothionamide, terizidone, thioacetazone, amoxicillin/clavulanate, imipenem, bedaquiline, and delamanid has not yet been assessed and may bring new candidates for repurposing tuberculostatic agents towards malaria. Moreover, the screening of anti-TB compounds with reported blood stage activity against hepatic *Plasmodium* infection may provide new insights into their possible application as multi-stage anti-plasmodial strategies.

Helminths, or parasitic worms, are responsible for a high burden of disease in developing countries [212]. Among helminths, those belonging to the nematodes phylum are at the basis of soil-transmitted helminth infections, LF, and onchocerciasis, whereas the platyhelminths phylum contains the parasitic worms that cause schistosomiasis and fascioliasis [212]. Soil-transmitted infections are the most common helminthiasis, affecting approximately 1.5 billion people worldwide, and are widely distributed in tropical and subtropical regions, overlapping with *Plasmodium* infections. To tackle this problem the WHO recommends periodical treatment with the inexpensive drugs albendazole and mebendazole [213]. While these drugs have not been evaluated for liver stage activity, they were shown to inhibit *P. falciparum*’s intraerythrocytic development in vitro, although at relatively high concentrations [214,215]. Albendazole’s efficacy against malaria was further tested in mouse and rat models of *P. berghei* infection with contradicting results. Although albendazole was not effective in *P. berghei*-infected mice, it reduced parasitaemia in infected rats [216], which was later suggested to result from an indirect effect of the compound on the hematopoietic system [217]. Although albendazole has been employed in malaria-related randomized human studies, it was always in the context of helminth-*Plasmodium* co-infections [218,219,220,221,222]. Given that helminthic infections may increase the individuals’ risk of malaria, these studies do not allow one to draw conclusions regarding the drug’s action on the *Plasmodium* parasite. LF, commonly known as elephantiasis, may result in severe disability and, consequently, social stigma. Towards the long-term goal of elimination of this helminthic disease, WHO recommends annual mass drug administration to at-risk populations. The recommended regimens vary with the co-endemicity with other filarial diseases, and include the already mentioned albendazole, as well as ivermectin [223], which is also the recommended treatment agent for onchocerciasis [224]. The anti-plasmodial activity of diethylcarbamazine citrate, which is also part of the LF chemoprevention package [223], remains to be assessed. The control of schistosomiasis includes large-scale treatment with praziquantel [225]. Like albendazole, praziquantel has only been employed in human studies in the context of helminth-*Plasmodium* co-infection [218,221]. Thus, its activity in a clinical setting remains to be characterized. Finally, no reports exist on the anti-plasmodial activity of triclabendazole, a drug approved for human use by the FDA in 2019, and recommended by the WHO for the treatment of fascioliasis [226]. Overall, the knowledge of the possible anti-plasmodial activity of anti-helminthic drugs is limited, particularly against the liver stage of infection. Further studies may reveal bonus effects in the employment of such compounds for the prevention and/or control of malaria.

The protozoan *Leishmania* parasites, which are transmitted by phlebotomine sandflies, cause leishmaniasis in humans, a disease that can present as visceral, cutaneous, and mucocutaneous leishmaniasis. The type of the disease, the *Leishmania* species involved, the geographical location, and the existence of concomitant pathologies in a given patient will determine the type of treatment to be administered. WHO-recommended treatment options include the injectable pentavalent antimonials meglumine antimoniate and sodium stibogluconate, amphotericin B deoxycholate, liposomal amphotericin B, paromomycin (aminosidine), and pentamidine isethionate, and the oral miltefosine [227]. Administration of sodium stibogluconate to *P. yoelii*-infected mice increased the severity of infection [228]. A heat-treated form of amphotericin B deoxycholate presented activity against both chloroquine-sensitive and -resistant *P. falciparum* strains in vitro, being more effective against ring stage parasites than against the later trophozoite and schizont stages [229]. Furthermore, amphotericin B deoxycholate and liposomal amphotericin B were shown to selectively lyse trophozoite-infected erythrocytes, although the latter required higher incubation periods [230]. Miltefosine was shown to inhibit the activity of *P. falciparum* phosphoethanolamine methyltransferase, as well as to impact this parasite’s blood stage development [231]. Although the potential of some of these drugs to be employed against blood stage *Plasmodium* infection has been described, their liver stage activity remains unknown. Further efforts towards this characterization may bring new insights to the possible employment of these drugs as multistage anti-plasmodial strategies.

The co-endemicity of malaria and other infectious diseases provides an opportunity for repurposing drugs from the latter to the former. Medicines currently employed in the management of TB, helminth infections and leishmaniasis have been mostly reported to possess blood stage activity, but their potential as prophylactic agents remain largely uncharacterized. Evaluation of these and other anti-infective medicines may reveal bonus effects, multistage activity, and ultimately contribute to the malaria prophylactic and therapeutic toolbox.

## 5. Conclusions and Summary of Future Perspectives

Drug repurposing is an increasingly popular approach in the development of therapeutic tools to combat diseases or conditions for which novel pharmacological solutions are required. Drug repositioning is particularly attractive when dealing with malaria and other neglected diseases that exert their burden in some of the poorest regions of the globe. In fact, it has been argued that one of the key barriers to malaria treatment and chemoprevention lies in the reluctance of the pharmaceutical industry to invest in the development of drugs that would offer only limited marketing prospects [232]. The cost reduction associated with repurposing existing compounds, as opposed to developing drugs de novo, may therefore help overcome some of the economical hurdles in the way of the expansion of the current anti-plasmodial pharmacological armamentarium.

In addition to constituting a reservoir for dormant forms of hypnozoite-forming *Plasmodium* parasite species, the liver stage of *Plasmodium* infection is an ideal but underexplored target for prophylactic interventions against malaria [233]. The scarcity of compounds that inhibit this obligatory phase of the parasite’s life cycle can be at least partly attributed to its clinical silent nature and to the difficulties in addressing it experimentally, which persisted for a long time. However, significant progress made over the last few decades has rendered the liver stage *Plasmodium* infection increasingly amenable to investigation, including the assessment of drug activity against hepatic parasites. A wide array of parasite and host models, including in vitro and in vivo systems, is now available to researchers [234], and new hepatic platforms for drug screening are currently being developed [235]. This ever-expanding toolbox renders the repurposing of drugs against the liver stage of *Plasmodium* infection especially appealing, as it permits screening the activity of large numbers of existing compounds against this phase of the parasite’s life cycle. Moreover, by enabling the evaluation of the hepatic stage activity of drugs known to inhibit other stages of the parasite’s life cycle, it significantly amplifies the possibility of identifying and developing much-sought-after compounds with multi-stage anti-plasmodial activity [236].

As outlined in the present review, the number of FDA-approved drugs with demonstrated activity against hepatic malaria parasites has been growing substantially. It is also safe to expect that this number will continue rising in the near future, as laboratories around the world invest their efforts into evaluating the ability of existing drugs to effectively inhibit *Plasmodium* parasite development in the liver. Such studies should, however, bear in consideration that drugs to tackle a disease such as malaria need to be not only widely available but also largely affordable, if they are to be used by those who need them most. The WHO’s EML includes a large number of drugs that address the minimum medicine needs for efficacious, safe, and cost–effective medicines for priority conditions [237]. This list includes several drugs employed to combat infectious diseases that are co-endemic with malaria, offering a privileged opportunity for repurposing of those drugs. However, the EML also lists medicines employed to treat a large array of NCDs or chronic diseases, including cancers, cardiovascular and respiratory diseases, and diabetes. NCDs may affect people of all age groups and countries, but their burden is more heavily felt in middle- to low-income countries, and has increased in sub-Saharan Africa over the past two decades [238]. Although the prevention of these diseases should be largely focused on reducing risk factors, their management inevitably involves the prolonged intake of medication. Thus, these compounds may also represent an opportunity for repurposing against concomitant *Plasmodium* infection, possibly providing a bonus effect in malaria-endemic settings. Nevertheless, it should be noted that drugs targeting NCDs likely act on a host rather than a parasite target, raising concerns about undesired side-effects. On the other hand, as with all repurposed drugs, the safety and toxicity of these compounds will have been ascertained, limiting these risks. Moreover, it should also be noted that targeting host factors to combat malaria may also offer an opportunity to circumvent the development of resistance by the parasite [239]. Hence, while drugs used to treat NCDs may display activity as anti-plasmodials, their potential use to combat malaria needs to be carefully evaluated in each case.

Despite recent progress in the quest for an effective vaccine against malaria, with a particular emphasis on vaccine candidates that target the liver stage of the *Plasmodium* life cycle [240,241,242,243], such a vaccine remains elusive. As such, the need for other strategies to prevent and treat malaria remains as pressing as ever. In this context, novel drugs that afford the efficient chemoprophylaxis, treatment, and radical cure of *Plasmodium* infection continue to be urgently needed. While this may entail the development of novel chemotypes for anti-plasmodial intervention, as recently reviewed in [244,245,246], drug repurposing offers a promising alternative to traditional drug development pipelines, and can help fast forward solutions to effectively combat this devastating disease.

## Figures and Tables

**Table 1:** Summary of drugs that have been tested against the liver and/or blood stages of *Plasmodium* infection.

Original Purpose	Drug	*Plasmodium* Liver Stage 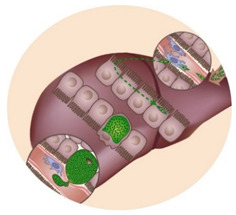					*Plasmodium* Blood Stage 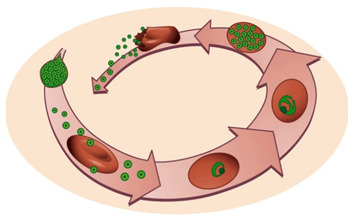								References
		Pb	Py	Pf	Pcy	Pk	Pb	Py	Pch	Pvin	Pf	Pv	Pcy	Pk	
**From HIV to Parasites**															
**Antiretroviral**	Saquinavir	x ^1^	x ^1^						x ^2^		x ^1^	x ^1^	x ^2^	x ^2^	[27,28,29,30,31,32,33,34,35]
	Lopinavir	x ^1^		x ^1^							x ^1^				[27,28,31,32,33,35,36]
	Ritonavir		x ^1^						x ^2^		x ^1^	x ^1^			[28,29,31,32,33,34,37]
	Indinavir		x ^1^								x ^1^		x ^2^	x ^2^	[27,28,30,32,34]
	Nelfinavir	x ^1,2^									x ^1^				[28,31,32,34,38]
	Lopinavir/Ritonavir		x ^2^		x ^2,3^		x ^2^		x ^2^		x ^1^				[28,37,39,40,41]
	Saquinavir/Ritonavir								x ^2^		x ^1^				[28,40]
	Efavirenz	x ^1^	x ^2^								x ^1^				[31,34,38,42]
	Etravirine	x ^1,2^	x ^2^								x ^1^				[31,34,38,42]
	Nevirapine		x ^2^				x ^2^				x ^1^				[31,34,42,43]
	Efavirenz/Zidovudine/Lamivudine	x ^2^													[38]
	Efavirenz/Tenofovir/Emtricitabine	x ^2^													[38]
	Nevirapine/Tenofovir/Emtricitabine	x ^2^													[38]
**From Bacteria to Parasites**															
Antibacterial	Demeclocycline				x ^2^								x ^2^		[44]
	Terramycin				x ^2^										[45]
	Minocycline	x ^1^			x ^2,3^		x ^2^								[46,47,48,49,50,51,52,53]
	Doxycycline	x ^1,2^	x ^1,2^		x ^2,3^						x ^1^				[48,53,54,55,56,57,58]
	Azithromycin	x ^2^	x ^2^		x ^2,3^		x ^2^				x ^1^				[48,53,56,59,60,61,62,63,64]
	Grepafloxacin		x ^1^	x ^1^							x ^1^				[65]
	Norfloxacin		x ^1^								x ^1^				[48,65,66,67,68]
	Piromidic acid		x ^1^	x ^1^							x ^1^				[65]
	Trovafloxacin		x ^1^	x ^1^							x ^1^				[65,67]
	Cinoxacin		x ^1^								x ^1^				[65]
	Ciprofloxacin		x ^1^		x ^2,3^			x ^2^			x ^1^				[53,56,65,66,67,68,69,70,71]
	Rufloxacin		x ^1^								x ^1^				[65]
	Sparfloxacin		x ^1^								x ^1^				[65]
	Ofloxacin		x ^1^								x ^1^				[48,65,66,68]
	Temafloxacin		x ^1^								x ^1^				[65]
	Pefloxacin		x ^1^								x ^1^				[65,66]
	Clinafloxacin		x ^1^								x ^1^				[65]
	Dapsone		x ^2^												[72]
	Co-trimoxazole	x ^1^	x ^1,2^	x ^1^	x ^2,3^	x ^2^					x ^1^				[41,42,73,74,75]
	Sulfadiazine	x ^2^	x ^2^				x ^2^				x ^1^		x ^2^		[72,76,77,78,79]
	Pyrazinamide				x ^2,3^										[53]
	Clindamycin				x ^2,3^										[53]
**Repurposing within Parasitism**															
Antiparasitic, insecticidal	Ivermectin	x ^1,2^									x ^1^				[47,80,81,82]
	Abamectin	x ^1^													[47]
	Emamectin	x ^1^													[80]
	Eprinomectin	x ^1^													[80]
Antitrypanosomal	Suramin			x ^1^			x ^2^				x ^1^				[83,84,85,86,87,88,89]
	Eflornithine (DFMO)	x ^1,2^					x ^2^				x ^1^				[90,91,92,93,94,95,96,97,98,99,100,101,102]
	Pentamidine						x ^1^				x ^1^				[103,104,105,106,107,108,109,110,111,112,113]
Antiprotozoal	Tinidazole				x ^2,3^										[53]
**From Chronicity to Infection**															
Antidiabetic	Metformin	x ^2^		x ^1^			x ^2^	x ^2^			x ^1^				[114,115]
Antihistaminic	Cyproheptadine		x ^2^				x ^2^	x ^2^			x ^1^				[116,117,118,119,120]
	Ketotifen		x ^2^				x ^2^	x ^2^			x ^1^				[116,117,118,119]
	Terfenadine		x ^2^					x ^2^							[116]
	Azatadine		x ^2^					x ^2^							[116,119]
	Loratadine		x ^2^					x ^2^							[116,119]
	Clemastine	x ^1^									x ^1^				[121,122]
	Astemizole	x ^1^						x ^2^		x ^2^	x ^1^				[121,123]
	Promethazine				x ^2,3^										[53]
Immunosuppressive	Cyclosporin A	x ^1,2^					x ^2^	x ^2^	x ^2^		x ^1^	x ^1^			[121,124,125,126,127,128,129,130]
Proton pump inhibitor	Omeprazole	x ^1^													[121] and references therein
	Esomeprazole	x ^1^													
Angiotensin receptor antagonist	Telmisartan	x ^1^													
Ovulation inducer	Clomiphene	x ^1^													
Anticancer	Daunorubicin	x ^1^													
	Doxorubicin	x ^1^													
	Idarubicin	x ^1^													
Antirheumatic	Auranofin	x ^1^													
Immunosuppressive	Mycophenolic acid	x ^1^													
Estrogen receptor modulator	Tamoxifen citrate	x ^1^													[47] and references therein
	Toremiphene citrate	x ^1^													
Antianginal	Perhexiline maleate	x ^1^													
Diuretic	Triamterene				x ^2,3^										[53]

Pb–*P. berghei*; Py–*P. yoelii*; Pf–*P. falciparum*; Pcy–*P. cynomolgi*; Pk–*P. knowlesi*; Pch–*P. chabaudi*; Pvin–*P. vinckei*; Pv–*P. vivax*. ^1^ Tested in vitro. ^2^ Tested in vivo. ^3^ Tested against parasite dormancy. Images depict the liver and blood stages of the *Plasmodium* life cycle in the mammalian host, respectively.
